# miR-224, miR-147b and miR-31 associated with lymph node metastasis and prognosis for lung adenocarcinoma by regulating PRPF4B, WDR82 or NR3C2

**DOI:** 10.7717/peerj.9704

**Published:** 2020-11-24

**Authors:** Yan Wang, Shengtao Shang, Kun Yu, Hongbin Sun, Wenduan Ma, Wei Zhao

**Affiliations:** 1 Department of Thoracic Surgery, China-Japan Union Hospital of Jilin University, Jilin, China; 2 Department of Thoracic Surgery, Baicheng Hospital of Traditional Chinese Medicine, Jilin, China

**Keywords:** Lung adenocarcinoma, Lymph node metastasis, Survival, miRNAs, mRNA, Bioinformatics

## Abstract

**Background:**

The present study is to screen lymph node metastasis-related microRNAs (miRNAs) in lung adenocarcinoma (LUAD) and uncover their underlying mechanisms.

**Methods:**

The miRNA microarray dataset was collected from the Gene Expression Omnibus database under accession number GSE64859. The differentially expressed miRNAs (DEMs) were identified using a t-test. Target genes of DEMs were predicted through the miRWalk2.0 database. The function of these target genes was annotated with the clusterProfiler and the Database for Annotation, Visualization and Integrated Discovery (DAVID) tools. Protein-protein interaction network was established using the STRING database to extract hub target genes. The expressions and associations with survival and lymph node metastasis of miRNAs and target genes were validated by analysis of The Cancer Genome Atlas (TCGA) dataset.

**Results:**

Eight DEMs were identified between lymph node metastasis and non-metastasis samples of GSE64859 dataset. miRNA-target gene pairs were predicted between six DEMs and 251 target genes (i.e. hsa-miR-224-PRPF4B, hsa-miR-147b-WDR82 and hsa-miR-31-NR3C2). The clusterProfiler analysis showed WDR82 was involved in the mRNA surveillance pathway, while the GO enrichment analysis using the DAVID database indicated PRPF4B participated in the protein phosphorylation and NR3C2 was related with the transcription, DNA-templated. WDR82 and PRPF4B may be hub genes because they could interact with others. Two DEMs (miR-31-5p and miR-31-3p) and 45 target genes (including PRPF4B and NR3C2) were significantly associated with overall survival. The expressions of miR-224 and miR-147b were validated to be upregulated, while WDR82, PRPF4B and NR3C2 were downregulated in lymph node metastasis samples of TCGA datasets compared with non-metastasis samples. Also, there were significantly negative expression correlations between miR-147b and WDR82, between miR-224 and PRPF4B, as well as between miR-31 and NR3C2 in LUAD samples.

**Conclusions:**

The present study identified several crucial miRNA-mRNA interaction pairs, which may provide novel explanations for the lymph node metastasis and poor prognosis for LUAD patients.

## Introduction

Lung cancer is the leading cause of cancer-related death worldwide, with the number of deaths estimated up to three million by 2035 (Didkowska et al., 2016). Lung adenocarcinoma (LUAD) is the most common histological subtype, accounting for almost half of all lung cancers (Cadioli et al., 2014). The poor prognosis of patients with LUAD has been extensively considered to be resulted from tumor cell metastasis and invasion (Dai et al., 2017; Jeong, Kim & Pyo, 2017). The lymph node is the early metastatic region followed by distal spread (Dai et al., 2017; Jeong, Kim & Pyo, 2017). Thus, exploration of the mechanisms of lymph node metastasis may be beneficial to diagnose the patients at the early stage and therewith may reduce the overall mortality rate.

Recently, numerous reports have suggested that aberrant expression of non-encoding RNAs (including microRNAs (miRNAs), circular RNAs (circRNAs), and long ncRNAs (lncRNAs)) contributes to carcinogenesis and progression (Anastasiadou, Jacob & Slack, 2018). Among these non-encoding RNAs, microRNAs (miRNAs), a class of small, non-coding RNAs of 18–25 nucleotides, may be especially important because 1) they are highly conserved; 2) they could regulate the expression of multiple target mRNAs via binding to their 3′-untranslated regions (3′-UTRs) and then causing mRNA degradation or translation inhibition and then affect cellular proliferation, apoptosis, metastasis and invasion; and 3) they could mediate the link between circRNAs/lncRNAs and mRNAs (Anastasiadou, Jacob & Slack, 2018; Yin et al., 2019; Ghafouri-Fard et al., 2020). Thus, miRNAs may be underlying targets for explaining the pathogenesis of lymph node metastasis of LUAD, which had been reported in some studies previously. For example, Xu et al. (2015) analyzed the differentially expressed miRNAs (DEMs) between five lung cancer cases with lymph node metastasis and four cases without metastasis using the microarray and found miR-1260b was significantly upregulated in metastasis than that in the non-metastasis group. Li et al., (2017) confirmed that miR-342-3p and miR-150-5p could be lymphatic metastasis-related miRNAs in LUAD based on microarray and real-time PCR analyses, and predicted that they may function by regulating the EP300 and EP30, respectively. Meng et al. (2013) used the miRNA-seq data analysis to identify that miR-31 and miR-224 were upregulated in LUAD tissues from patients with lymph node metastases compared to those without lymph node metastases. miR-31 (Meng et al., 2013) and miR-224 (Cui et al., 2015) promoted tumor metastasis by influencing epithelial-mesenchymal transition (EMT) (Vimentin, TWIST1 and SNAI1) and TGF-*β* signaling genes (TNFAIP1 and SMAD4), respectively. However, data on the contributions of miRNAs in LUAD lymph node metastasis remain limited.

The present study is to further screen crucial miRNAs and explore their mechanisms for lymph node metastasis in LUAD by using the dataset of Meng et al. (2013) and combining with the survival outcomes.

## Materials and Methods

### Microarray data

The miRNA profile dataset was collected from the Gene Expression Omnibus (GEO) database (http://www.ncbi.nlm.nih.gov/geo/) under accession number GSE64859 (Cui et al., 2015; Meng et al., 2013). This dataset included lung tissue samples from 10 LUAD patients, among whom 4 exhibited lymph node metastasis (N1 stage) and 6 did not have lymph node metastasis (N0 stage). miRNA profile was determined by using high-throughput sequencing on AB SOLiD 4 System.

### Data preprocessing and differential analysis

The raw count matrix data were downloaded from the GPL13393 platform and preprocessed using the edgeR package (v3.22.3; http://bioconductor.org/help/search/index.html?q=edgeR/) in R (Robinson, McCarthy & Smyth, 2010). The DEMs between LUAD samples with and without lymph node metastasis were identified using the Linear Models for Microarray Data (LIMMA) method (v2.16.4; http://bioconductor.org/packages/release/bioc/html/limma.html) and *t*-test statistic (Ritchie et al., 2015). The false discovery rate (FDR) < 0.05 and —log fold change (FC)— > 2 were considered to be the cut-off point. A principal component analysis (PCA) was performed to determine whether the identified DEGs could classify the samples into metastasis and non-metastasis groups.

### Target gene prediction for DEMs

mRNA targets of DEMs were predicted using the miRWalk database (v2.0; http://zmf.umm.uni-heidelberg.de/apps/zmf/mirwalk2/) based on the search condition: minimum seed length = 7, *p* < 0.05 and input parameters = 3′-UTR. Only the miRNA-target gene interaction pairs that were predicted by at least any 9 algorithms of 12 in the miRWalk database (DIANA-microTv4.0, DIANA-microT-CDS, miRanda-rel2010, mirBridge, miRDB4.0, miRmap, miRNAMap, PicTar2, PITA, RNA22v2, RNAhybrid2.1 and Targetscan6.2) were collected to construct the miRNA-mRNA regulatory network. The network was visualized by the Cytoscape software (v2.8; http://www.cytoscape.org/) (Kohl, Wiese & Warscheid, 2011).

### Functional enrichment analysis

Kyoto Encyclopedia of Genes and Genomes (KEGG) pathway enrichment analysis was performed for target genes of each miRNA using the clusterProfiler tool (v3.2.11; http://www.bioconductor.org/packages/release/bioc/html/clusterProfiler.html) (Thissen, Steinberg & Kuang, 2002), with adjusted *p*-value < 0.05 set as the cut-off value. In addition, Gene Ontology (GO) biological process terms, KEGG and REACTOME_PATHWAY were also predicted using all the target genes as the seeds via searching the Database for Annotation, Visualization and Integrated Discovery (DAVID) database (v6.8; http://david.abcc.ncifcrf.gov) (Glynn Dennis et al., 2003). *P* < 0.05 and count > 2 were defined as the statistical threshold for DAVID analysis.

### PPI network construction for the target genes of DEMs

The target genes were mapped into the STRING (Search Tool for the Retrieval of Interacting Genes; https://string-db.org/) database (Szklarczyk et al., 2015) to obtain their interaction relationships. The interaction pairs with high confidence (score > 0.9) were retrieved to construct the PPI network and visualized using the Cytoscape software (v2.8; http://www.cytoscape.org/) (Kohl, Wiese & Warscheid, 2011). The topological characteristic of degree centrality (the number of interactions pairs) was calculated for each protein to represent their scores in the PPI network. Genes with a higher degree score may be hub genes for diseases.

### Survival analysis and expression validation

The miRNA and mRNA sequence data (Level 3) of samples with LUAD and having survival information were downloaded from The Cancer Genome Atlas (TCGA; https://tcga-data.nci.nih.gov/, updated to February 2020). Survival curves of miRNAs and their target genes were generated using the Kaplan–Meier method, and a two-sided log-rank test was performed to assess the statistical differences in overall survival (OS) between the high-expression (>median expression level) and low-expression (≤ median expression level) groups using the survival package in R (v2.4; https://cran.r-project.org/web/packages/survival/index.html). Furthermore, the expressions of miRNAs and their target genes between lymph node metastasis and non-metastasis samples were further validated via an unpaired *t*-test. The associations between miRNAs and their target genes were confirmed by Pearson’s correlation analysis. *P* < 0.05 indicated a statistical difference.

## Results

### Identification of DEMs

**Figure 1:**
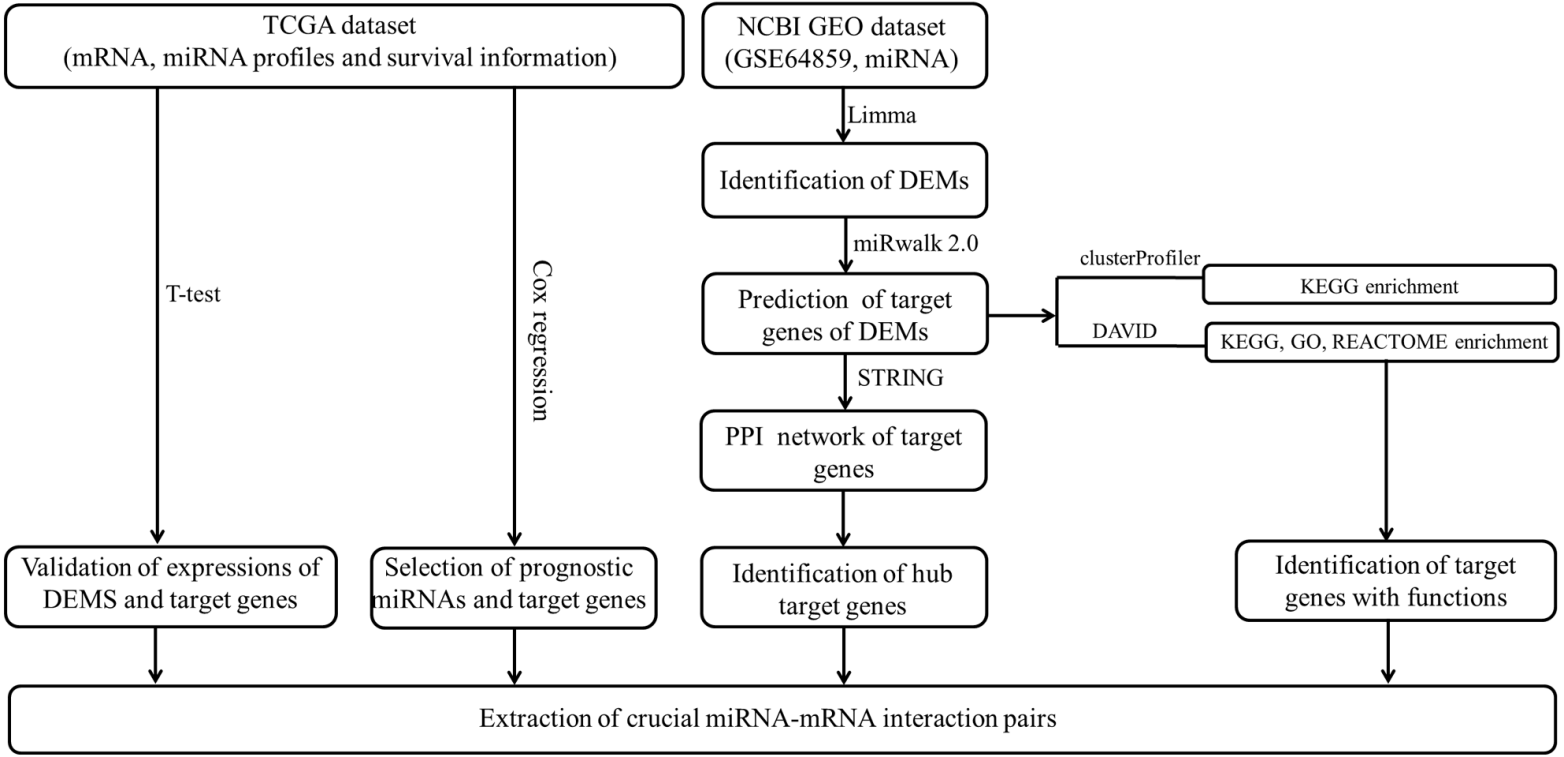
The data analysis workflow.

**Figure 2:**
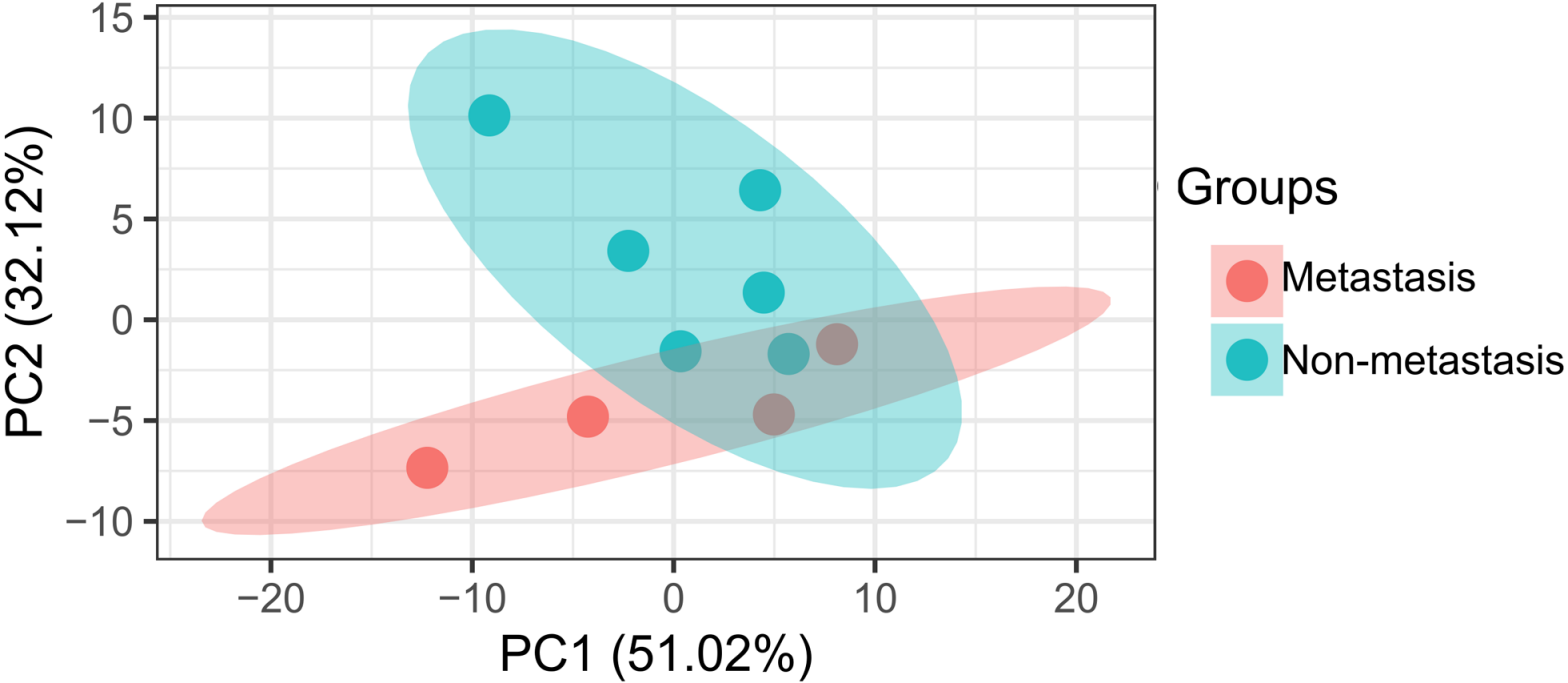
Principal component analysis of differentially expressed miRNAs in patients with lymph node metastasis compared to those without lymph node metastasis.

The data analysis workflow is shown in Fig. 1. Analysis of miRNA expression profiles in LUAD tissues from patients with lymph node metastasis compared to those without lymph node metastasis identified a total of 8 DEMs (Table 1). Of them, 6 miRNAs were upregulated (including hsa-miR-3975, hsa-miR-1269a, hsa-miR-224-5p, hsa-miR-147b, hsa-miR-31-5p and hsa-miR-31-3p), while 2 were downregulated (including hsa-miR-371b-5p and hsa-miR-371a-3p). The PCA indicated these DEMs could well classify the samples into metastasis and non-metastasis groups (Fig. 2).

**Table 1:** Differentially expressed miRNAs.

miRNA	logFC	*P*-value	FDR
hsa-miR-3975	5.57	1.82E−05	2.73E−03
hsa-miR-1269a	4.84	2.15E−04	1.93E−02
hsa-miR-31-3p	4.76	1.65E−04	1.86E−02
hsa-miR-224-5p	4.40	3.46E−04	2.59E−02
hsa-miR-147b	4.29	4.58E−04	2.94E−02
hsa-miR-31-5p	4.09	7.38E−04	4.14E−02
hsa-miR-371b-5p	−7.37	1.37E−05	2.73E−03
hsa-miR-371a-3p	−7.37	1.37E−05	2.73E−03

**Notes.**

FC fold change FDR false discovery rate

### Construction of a DEM–target gene regulatory network

**Figure 3:**
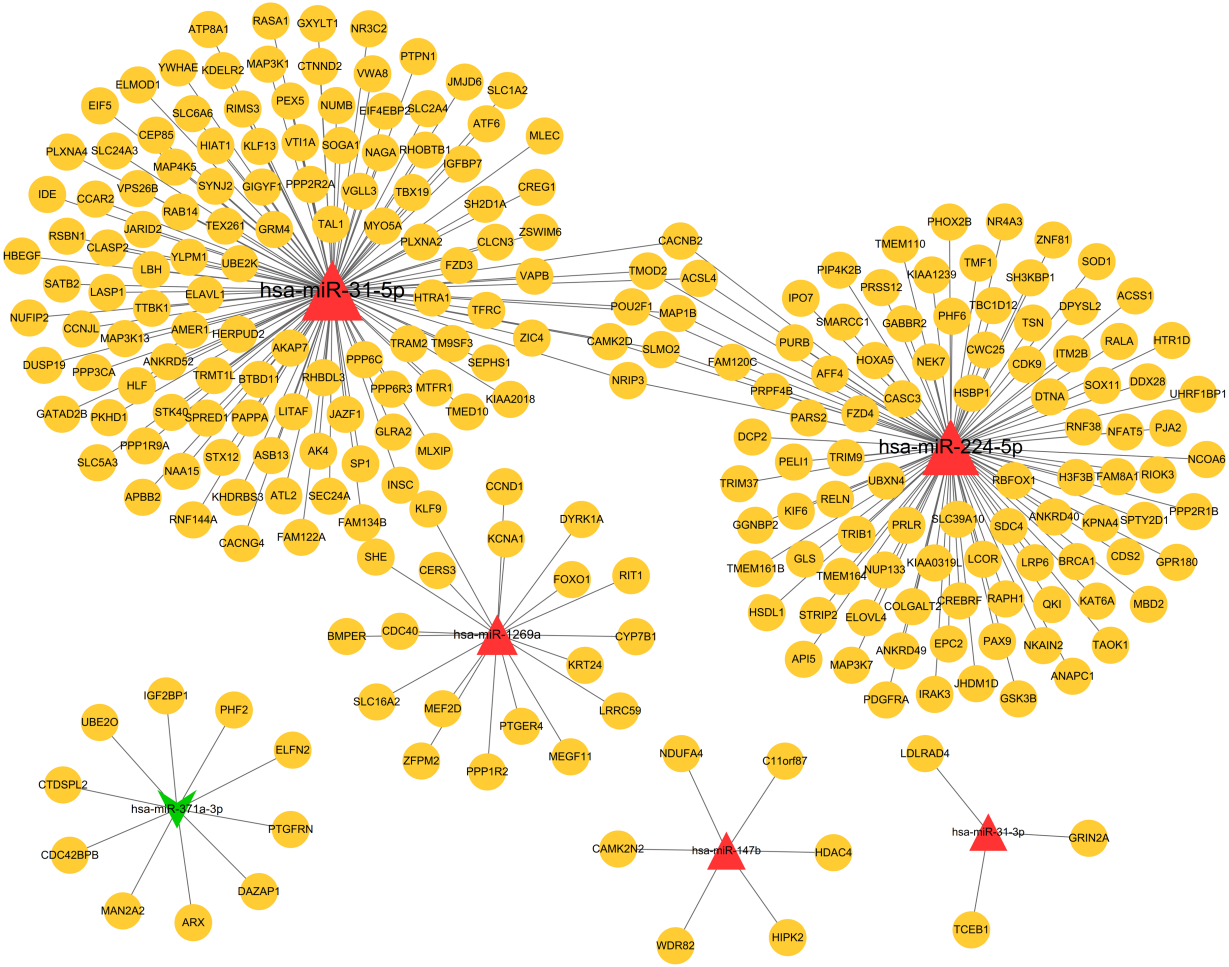
The miRNA-target genes regulatory network. Red, upregulated miRNAs; green, downregulated miRNAs; the circular indicated target genes.

Based on the given threshold value (predicted by more than 8 algorithms of 12 in the miRWalk 2.0 database), a total of 259 miRNA–target gene interaction pairs (such as hsa-miR-371a-3p-PTGFRN) were predicted, including 6 DEMs and 251 target genes, which were used for constructing the miRNA–target regulatory network (Table S1; Fig. 3).

### Function enrichment for the target genes in the miRNA regulatory network

**Figure 4:**
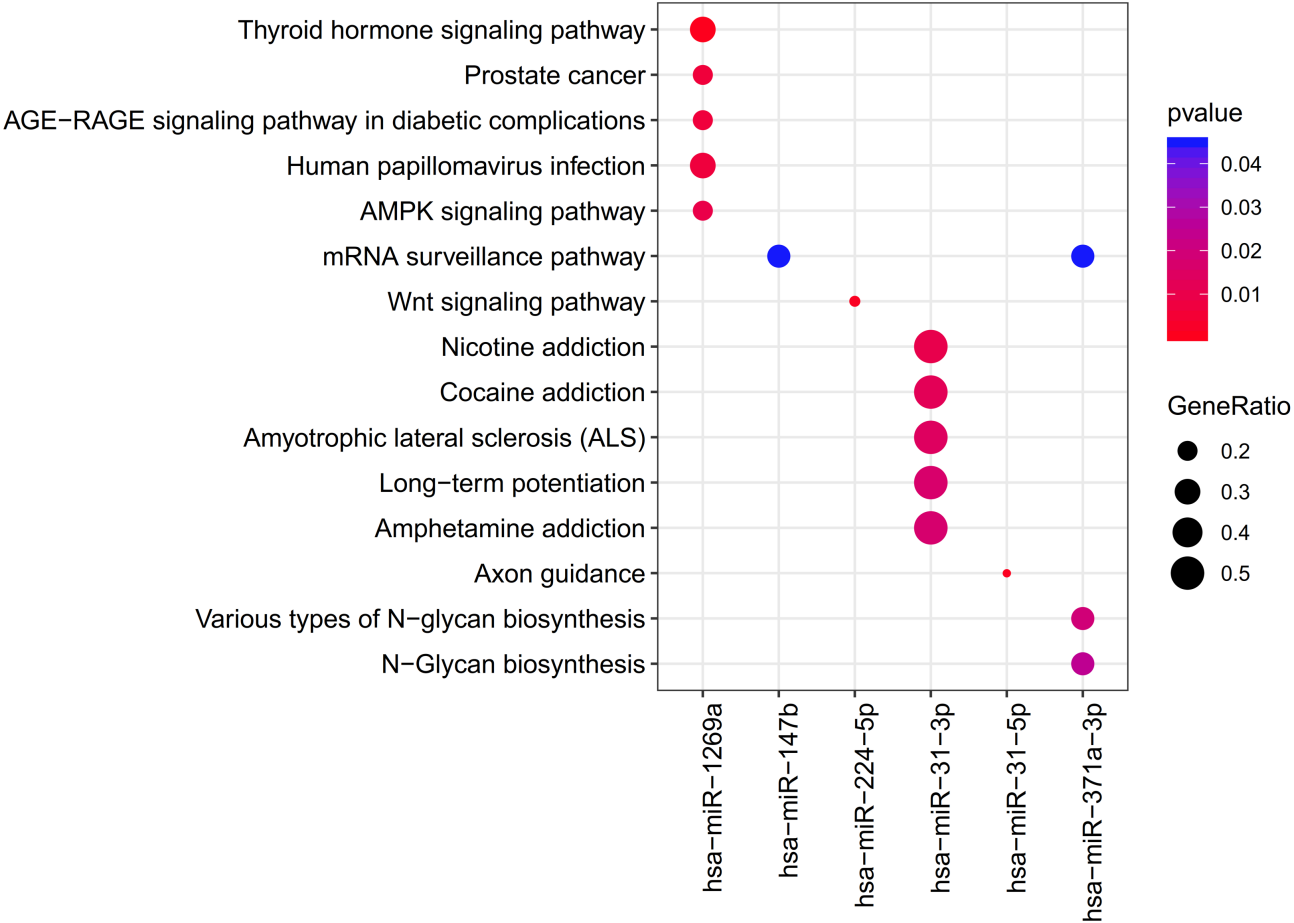
The clusterProfiler for prediction of the KEGG pathways of each miRNA.

The clusterProfiler was used for the prediction of the KEGG pathways of each miRNA. The results showed that the target genes of 6 DEMs were enriched to 33 significant pathways, including 11 for hsa-miR-1269a (such as Human papillomavirus infection; PTGER4 (prostaglandin E receptor 4)), 1 for hsa-miR-147b (mRNA surveillance pathway; WDR82 (WD repeat domain 82)), 1 for hsa-miR-224-5p (Wnt signaling pathway), 16 for hsa-miR-31-3p (such as Amphetamine addiction, Calcium signaling pathway; GRIN2A (glutamate ionotropic receptor NMDA type subunit 2A)), 1 for hsa-miR-31-5p (Axon guidance) and 3 for hsa-miR-371a-3p (mRNA surveillance pathway, DAZAP1(DAZ associated protein 1)) (Table 2; Fig. 4).

**Table 2:** KEGG enrichment for the genes of differentially expressed miRNAs.

miRNA	ID	Description	Adjusted *p*-value	Gene ID
hsa-miR-1269a	hsa04919	Thyroid hormone signaling pathway	3.60E−04	CCND1/FOXO1/SLC16A2
hsa-miR-1269a	hsa05215	Prostate cancer	6.18E−03	CCND1/FOXO1
hsa-miR-1269a	hsa04933	AGE-RAGE signaling pathway in diabetic complications	6.56E−03	CCND1/FOXO1
hsa-miR-1269a	hsa05165	Human papillomavirus infection	6.78E−034	CCND1/FOXO1/PTGER4
hsa-miR-1269a	hsa04152	AMPK signaling pathway	9.33E−03	CCND1/FOXO1
hsa-miR-1269a	hsa04068	FoxO signaling pathway	3.60E−02	CCND1/FOXO1
hsa-miR-1269a	hsa04371	Apelin signaling pathway	1.20E−02	CCND1/MEF2D
hsa-miR-1269a	hsa04218	Cellular senescence	1.62E−02	CCND1/FOXO1
hsa-miR-1269a	hsa00120	Primary bile acid biosynthesis	2.11E−02	CYP7B1
hsa-miR-1269a	hsa05163	Human cytomegalovirus infection	3.07E−02	CCND1/PTGER4
hsa-miR-1269a	hsa05216	Thyroid cancer	4.54E−02	CCND1
hsa-miR-147b	hsa03015	mRNA surveillance pathway	4.49E−02	WDR82
hsa-miR-224-5p	hsa04310	Wnt signaling pathway	1.28E−03	CAMK2D/GSK3B/LRP6/ MAP3K7/FZD4
hsa-miR-31-3p	hsa05033	Nicotine addiction	1.00E−02	GRIN2A
hsa-miR-31-3p	hsa05030	Cocaine addiction	1.22E−02	GRIN2A
hsa-miR-31-3p	hsa05014	Amyotrophic lateral sclerosis (ALS)	1.42E−02	GRIN2A
hsa-miR-31-3p	hsa04720	Long-term potentiation	1.67E−02	GRIN2A
hsa-miR-31-3p	hsa05031	Amphetamine addiction	1.72E−02	GRIN2A
hsa-miR-31-3p	hsa05211	Renal cell carcinoma	1.72E−02	ELOC
hsa-miR-31-3p	hsa04713	Circadian entrainment	2.42E−02	GRIN2A
hsa-miR-31-3p	hsa05017	Spinocerebellar ataxia	2.44E−02	GRIN2A
hsa-miR-31-3p	hsa04066	HIF-1 signaling pathway	2.71E−02	ELOC
hsa-miR-31-3p	hsa04724	Glutamatergic synapse	2.84E−02	GRIN2A
hsa-miR-31-3p	hsa04728	Dopaminergic synapse	3.26E−02	GRIN2A
hsa-miR-31-3p	hsa05322	Systemic lupus erythematosus	3.30E−02	GRIN2A
hsa-miR-31-3p	hsa04120	Ubiquitin mediated proteolysis	3.38E−02	ELOC
hsa-miR-31-3p	hsa05010	Alzheimer disease	4.24E−02	GRIN2A
hsa-miR-31-3p	hsa05034	Alcoholism	4.56E−02	GRIN2A
hsa-miR-31-3p	hsa04020	Calcium signaling pathway	4.78E−02	GRIN2A
hsa-miR-31-5p	hsa04360	Axon guidance	9.68E−04	FZD3/CAMK2D/PLXNA2/ PPP3CA/RASA1/PLXNA4
hsa-miR-371a-3p	hsa00513	Various types of N-glycan biosynthesis	1.94E−02	MAN2A2
hsa-miR-371a-3p	hsa00510	N-Glycan biosynthesis	2.48E−02	MAN2A2
hsa-miR-371a-3p	hsa03015	mRNA surveillance pathway	4.49E−02	DAZAP1

To further investigate the underlying functions of the target genes in the miRNA regulatory network, DAVID enrichment analysis was performed for all target genes. As a result, eight KEGG pathways were enriched, including hsa05014:Amyotrophic lateral sclerosis (ALS) (SLC1A2 (solute carrier family 1 member 2), GRIN2A); 13 REACTOME pathways were obtained, including R-HSA-399956: CRMPs in Sema3A signaling (DPYSL2 (dihydropyrimidinase like 2)); and 60 GO biological process terms were identified, containing GO:0006468 ∼protein phosphorylation (PRPF4B [pre-mRNA processing factor 4B]), GO:0006351 ∼transcription, DNA-templated (NR3C2 (nuclear receptor subfamily 3 group C member 2)), GO:0007268 ∼chemical synaptic transmission (SLC1A2), GO:0007399∼nervous system development (DPYSL2) and GO:0048208 ∼COPII vesicle coating (PPP6C) (Table S2). Accordingly, these genes-related miRNA-mRNA regulatory pairs (miR-224-5p-PRPF4B/DPYSL2, miR-31-5p-SLC1A2/NR3C2/PPC6) may also be crucial.

### Construction of a PPI network for the target genes of DEMs

**Figure 5:**
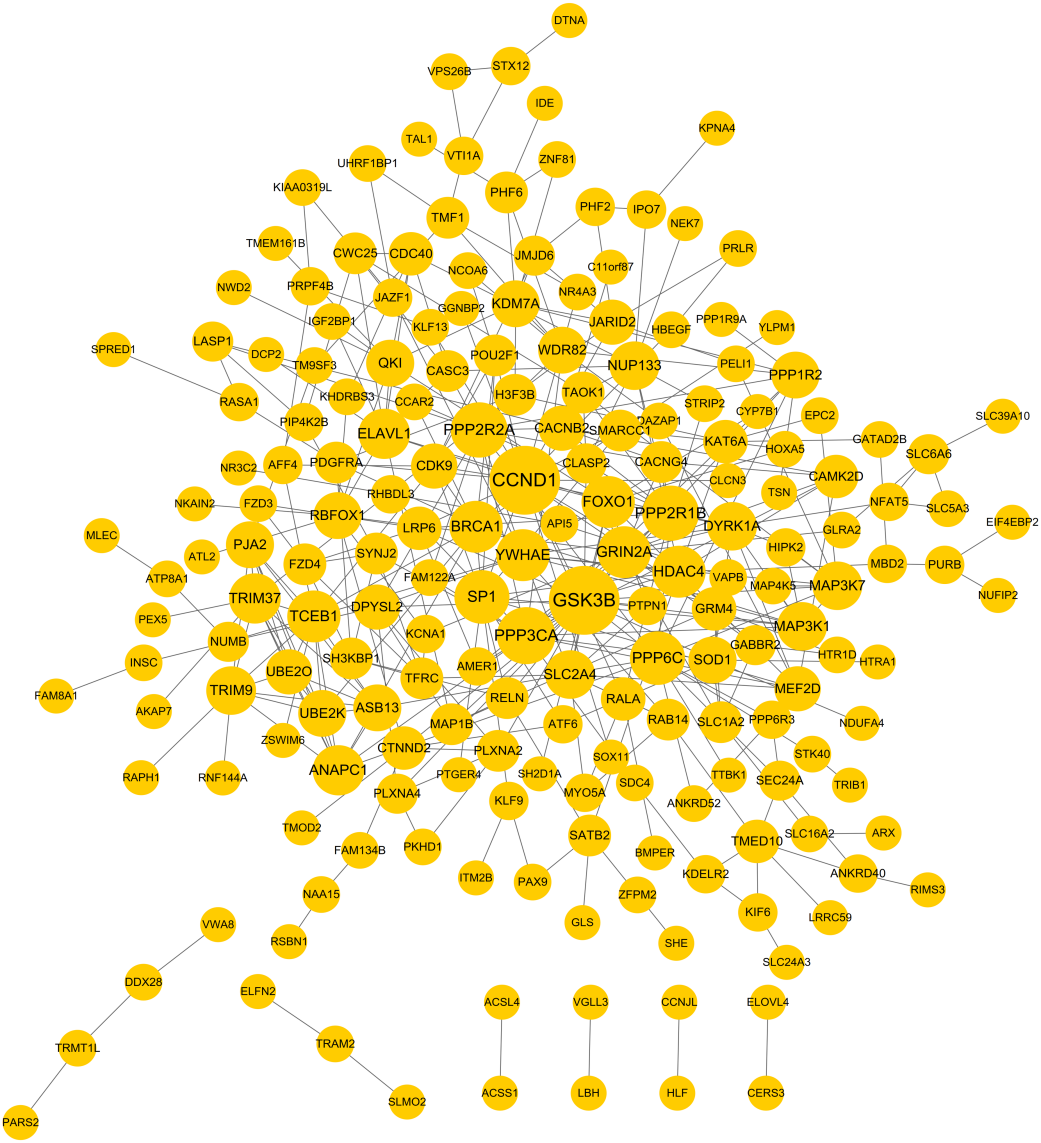
Protein and protein interaction (PPI) network for the target genes of differentially expressed miRNAs.

PPI network was constructed to further screen the hub target genes. By searching the STRING database, 381 interaction relationships (Table S3) were found to be present in 196 target genes of DEMs, which were used to construct the PPI network (such as WDR82-PPP2R2A) (Fig. 5). The function-enriched PPP6C (degree = 12), GRIN2A (degree = 11), WDR82 (degree = 8), DPYSL2 (degree = 7), SLC1A2 (degree = 5) and PRPF4B (degree = 3) were found to have a relatively high degree score (Table S3), further indicating their hub roles for LUAD.

### Identification of survival or lymph node metastasis-related target genes of DEMs

**Figure 6:**
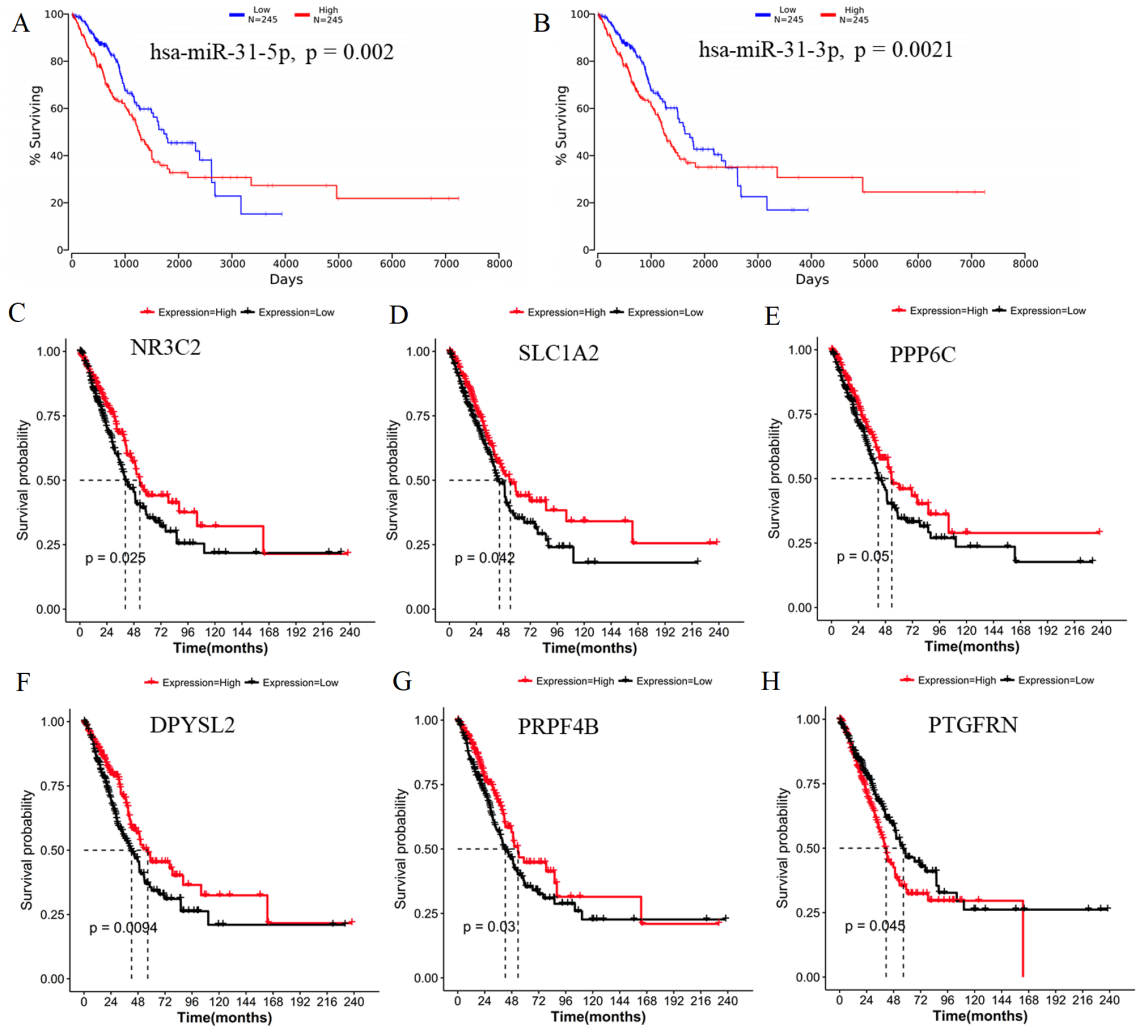
Survival analysis using the data from the Cancer Genome Atlas (TCGA) database. (A) miR-31-5p; (B) miR-31-3p; (C) NR3C2; (D) SLC1A2; (E) PPP6C; (F) DPYSL2; (G) PRPF4B; (H) PTGFRN.

**Figure 7:**
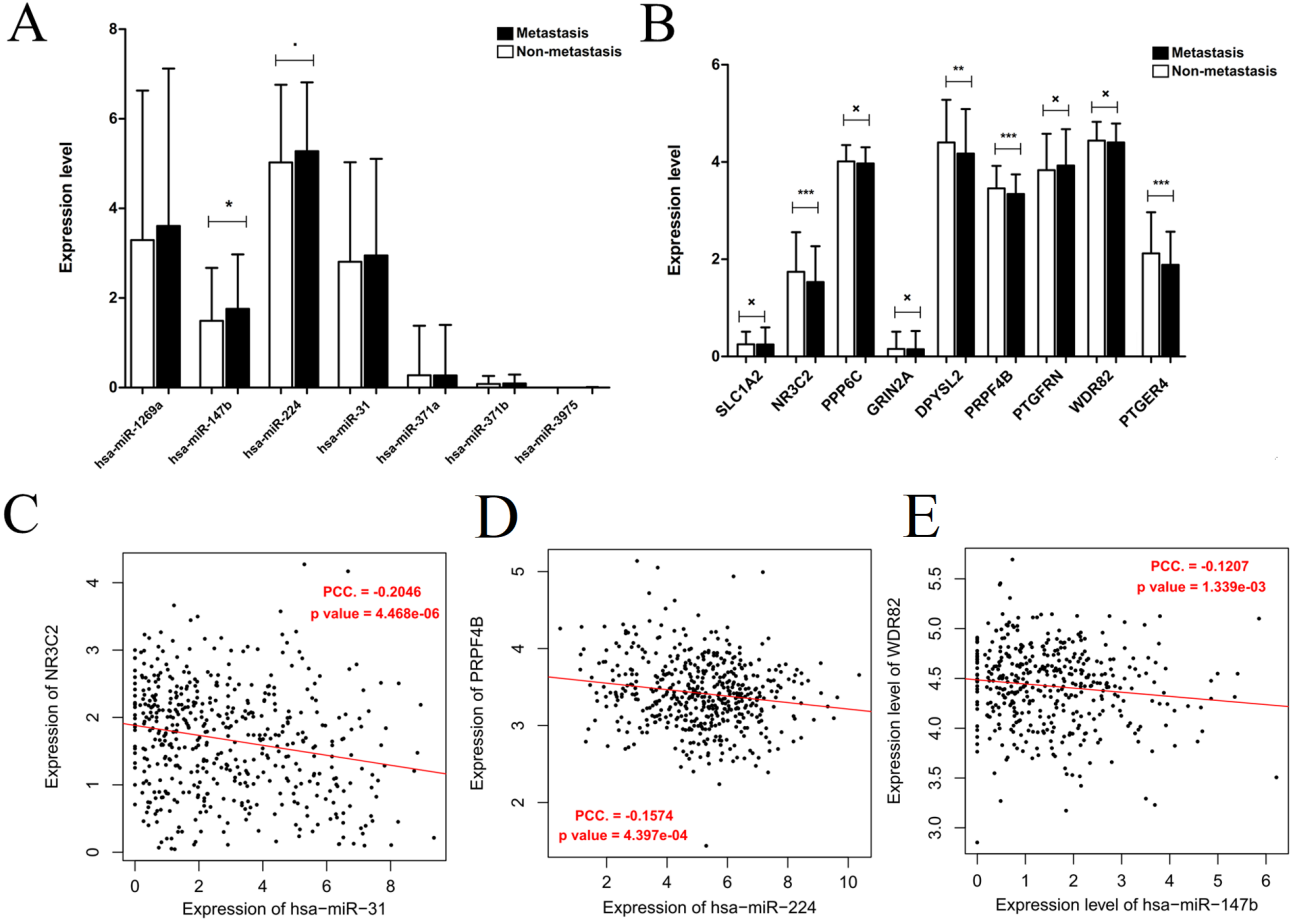
Expression and correlation validation with the data from the Cancer Genome Atlas (TCGA) database. (A) The expressions of miRNAs in lymph node metastasis samples (N1 + N2 + N3) compared with non-metastasis (N0); (B) the expressions of target genes in lymph node metastasis samples (N1 + N2 + N3) compared with non-metastasis (N0); (C) the expression correlation between miR-31 and NR3C2; (D) the expression correlation between miR-224 and PRPF4B; (E) the expression correlation between miR-147b and WDR82. The expression correlation between hsa-miR-147b and WDR82 was validated in N1 and N0 samples, while the other in N0 + N1 + N2 + N3 samples.

Generally, patients with the local lymph node metastasis have a poor prognosis. Thus, survival analysis was also performed for our identified DEMs and their target genes using the TCGA data. The results revealed that only two DEMs (hsa-miR-31-5p and hsa-miR-31-3p) and 45 of 251 target genes (including DPYSL2, PRPF4B, SLC1A2, NR3C2, PPP6C and PTGFRN) were found to be significantly associated with OS (Fig. 6; Table S4). Furthermore, using the TCGA data of LUAD (supplementary Table S5), we validated that the expressions of hsa-miR-224 and hsa-miR-147b were upregulated (Fig. 7A), while DPYSL2, PRPF4B, NR3C2 and PTGER4 (Fig. 7B) were significantly downregulated in lymph node metastasis samples (including N1 + N2 + N3 stage, *n* = 169) compared with non-metastasis samples (N0 stage, *n* = 326). In addition, the expression of WDR82 was lower in the stage N1 group (*n* = 93) than that of the stage N0 samples with a marginal difference (*p* = 0.07). Also, there were significantly negative associations between the expression level of miR-224 and PRPF4B (*r* =  − 0.1574, *p* = 4.397E−04), between miR-31 and NR3C2 (*r* =  − 0.2046, *p* = 4.468E−06) as well as between hsa-miR-147b and WDR82 (*r* =  − 0.1207, *p* = 1.339E−03) in LUAD samples (Fig. 7C).

## Discussion

In the present study, by re-analysis of the microarray dataset of GSE64859 (Cui et al., 2015; Meng et al., 2013), we identified eight lymph node metastasis-related DEMs. The expressions of two DEMs (hsa-miR-224 and hsa-miR-147b, both upregulated) were further validated using the TCGA dataset. The target genes of them were demonstrated to be negatively associated with hsa-miR-224 (PRPF4B) and hsa-miR-147b (WDR82), showing downregulated expressions in lymph node metastasis samples of TCGA dataset. Also, they were significantly associated with OS. Furthermore, both of miR-31 and its target genes (NR3C2) were proved to be OS-related. Similarly, their expressions were negatively correlated. These findings suggested hsa-miR-224-PRPF4B, hsa-miR-147b-WDR82 and hsa-miR-31-NR3C2 may be experimentally verifiable interaction pairs to promote lymph node metastasis and poor prognosis of LUAD patients.

Our differential analysis results on hsa-miR-224 and hsa-miR-31 were in line with the studies of Meng (Meng et al., 2013) and Cui (Cui et al., 2015), both showing their upregulated expressions in lymph node metastasis samples of LUAD. However, the focused downstream targets of hsa-miR-224 and hsa-miR-31 in our study were different from the study of Meng (hsa-miR-31: EMT genes) (Meng et al., 2013) and Cui (hsa-miR-224: TNFAIP1 and SMAD4) (Cui et al., 2015), indicating the novel mechanisms to explaining the pro-metastatic roles of hsa-miR-224 and hsa-miR-31. Although the interaction relationships between hsa-miR-224 and PRPF4B, as well as between hsa-miR-31 and NR3C2/PPP6C have not been experimentally demonstrated previously, the function studies on these target genes in cancers may indirectly reveal the possible roles of hsa-miR-224 and hsa-miR-31. Liu et al. found that PRPF4B was significantly downregulated in hepatocellular carcinoma tissues compared with matched non-tumor tissues. Knockdown of PRPF4B enhanced cell viability, promoted cell proliferation, colony formation and facilitated the G1/S-phase transition of QGY-7703 and HepG2 cell lines (Liu et al., 2013). Corkery et al. (2015) demonstrated shRNA-mediated silencing of PRPF4B resulted in a significantly decreased sensitivity of breast and ovarian cancer cell lines to paclitaxel, which was also confirmed in the study of Lahsaee et al. (2016). Subsequent in vivo mouse model study of Corkery et al. (2018) further showed that loss of PRPF4B promoted the metastasis of ovarian cancer cells to the diaphragm, peritoneal wall and ascitic fluid. Mechanistic investigation indicated depletion of PRPF4B may exert pro-metastatic effects by reducing the degradation of epidermal growth factor receptor and increasing the expression of vimentin (Corkery et al., 2018). Patients with positive or high PRPF4B expression had better overall disease-free survival and OS than those with negative or low expression (Corkery et al., 2015; Corkery et al., 2018). Similarly, NR3C2 expression was also found to be reduced in patients with pancreatic cancer (Yang et al., 2016), clear-cell renal cell carcinoma (Zhao et al., 2018) and colon adenocarcinoma (Yu et al., 2019). Lower NR3C2 expression was correlated with T status, histological grade and poor OS (Yang et al., 2016; Yu et al., 2019; Zhao et al., 2018). Functional analysis showed that NR3C2 inhibition may promote pancreatic cancer cell metastasis by inducing EMT, with a reduction in E-cadherin and an increase in zeb1, N- cadherin and vimentin at mRNA and protein levels (Yang et al., 2016; Zhang et al., 2017). Genome-wide lethality screening in non-small cell lung cancer cells also reported that NR3C2 may be a potential tumor-suppressing gene (Zhang et al., 2018). In accordance with these studies, we also found PRPF4B and NR3C2 were downregulated in metastasis samples and the OS rate in patients with high expression of PRPF4B or NR3C2 was higher than that in patients with low expression of them in LUAD.

Although Meng et al. (2013) and Cui et al. (2015) also identified miR-147b as a lymph node metastasis-related DEM, they did not explore its function mechanisms. The association of miR-147b with metastasis in lung cancer was also rarely investigated except for a recent study performed by Feng et al. (2020). Feng et al. observed miR-147b was up-regulated in LUAD tissues and cell lines, which induced poor prognosis outcomes. *In vitro* experiments revealed that miR-147b could promote cell proliferation, colony formation, invasion and migration. Mechanism analysis showed microfibril-associated glycoprotein 4 may be the putative target gene of miR-147b (Feng et al., 2020). In this study, we predicted a new possible target (WDR82) to explain the pro-metastatic mechanisms for miR-147b in LUAD. The function of WDR82 and its family genes in cancers may indirectly verify our speculation. For example, the immunohistochemistry results of Liu et al. demonstrated that the expression level of WDR82 was significantly decreased in colorectal cancer tissues compared with paired non-cancerous tissues. The low expression level of WDR82 was associated with lymph node, liver metastasis and reduced OS (Liu et al., 2018). PCR, immunoblotting, and immunohistochemistry analyses also found that WDR34 was downregulated in oral cancer compared with normal control tissues (Yamamoto et al., 2018). Overexpression of WDR34 *in vitro* depressed tumoral growth (Yamamoto et al., 2018). WDR76 deficient mice were reported to develop more tumors with bigger sizes than control mice and their tumors showed increased proliferation and cancer stem cell activation (Ro et al., 2019). A lower level of WDR76 was associated with poor survival of colorectal cancer patients (Ro et al., 2019). WDR76 functioned as a tumor suppressor mainly via degradation of RAS and then deactivation of Wnt/*β*-catenin signaling (Ro et al., 2019). Using the TCGA dataset, Takahashi et al. screened that downregulated WDR20 was significantly associated with poorer outcomes in patients with clear cell renal cell carcinoma. Subsequent functional assays showed that exogenous WDR20 significantly inhibited the growth of renal carcinoma cells and induced apoptosis, which may be resulted from the deactivation of downstream ERK and protein kinase B/AKT signaling pathways (Takahashi et al., 2016). Consistent with these studies, we also found WDR82 was lowly expressed in metastasis samples. Also, WDR82 was predicted to interact with PPP2R2A; while PPP2R2A was reported to mediate inhibition of ERK and Akt pathways in cancer (Wang et al., 2016; Koutsioumpa et al., 2018).

In conclusion, the present study identified three crucial miRNAs (hsa-miR-224, hsa-miR-147b and hsa-miR-31) for diagnosing the patients with lymph node metastasis. Their interactions with target genes (hsa-miR-224-PRPF4B, hsa-miR-147b-WDR82 and hsa-miR-31-NR3C2) may reveal a novel explanation for the lymph node metastasis and poor prognosis and provide potential therapeutic targets for LUAD. However, there were some limitations to this study. First, the regulatory relationships between miRNAs and mRNAs were only predicted and confirmed by correlation analysis with high-throughput data. Dual-luciferase reporter assay, miRNA mimics or inhibitor transfection *in vitro* and in vivo experiments should be conducted to further elucidate their regulatory mechanisms. Second, PCR, immunoblotting, and immunohistochemistry analyses should be performed to verify the expressions of miRNAs and mRNAs and re-investigate their prognostic values, especially the miRNAs and mRNAs with negative results. Third, the other mechanisms (such as mutation) (Galka-Marciniak et al., 2019) influencing the interactions between miRNAs and mRNAs may also be future research directions.

##  Supplemental Information

10.7717/peerj.9704/supp-1 Table S1 miRNA–target gene interaction pairs

10.7717/peerj.9704/supp-2 Table S2 Function enrichment analyses by the DAVID database

10.7717/peerj.9704/supp-3 Table S3 Protein-protein interaction pairs and the degree calculation

10.7717/peerj.9704/supp-4 Table S4 Genes associated with OS

10.7717/peerj.9704/supp-5 Table S5 The expressions of validated miRNAs and target genes

## Additional Information and Declarations

### Data Availability

The following information was supplied regarding data availability: Data are available at NCBI GEO: GSE64859 and at The Cancer Genome Atlas under search term TCGA-LUAD.
